# Tissue-Specific Biomarkers and Bioaccumulation in *Mytilus galloprovincialis*: Seasonal Anthropogenic Stress in the North Ionian Sea (Calabria, Italy)

**DOI:** 10.3390/jox16030104

**Published:** 2026-06-04

**Authors:** Maria Assunta Iovine, Mariacristina Filice, Luisa Albarano, Alessia Caferro, Sandra Imbrogno, Rosa Mazza, Francesca Esposito, Maria Costantini, Valerio Zupo, Alfonsina Gattuso, Giovanni Libralato, Maria Carmela Cerra

**Affiliations:** 1 Department of Biology, Ecology and Earth Sciences, University of Calabria, 87036 Rende, Italy; mariassunta.iovine@gmail.com (M.A.I.); mariacristina.filice@unical.it (M.F.); alessia.caferro1@gmail.com (A.C.); sandra.imbrogno@unical.it (S.I.); rosa.mazza@unical.it (R.M.); maria_carmela.cerra@unical.it (M.C.C.); 2 Department of Biology, University of Naples Federico II, Complesso Universitario di Monte Sant’Angelo, Via Cinthia n.21, 80126 Naples, Italy; luisa.albarano@unina.it (L.A.); francesca.esposito@unina.it (F.E.); giovanni.libralato@unina.it (G.L.); 3 Department of Ecosustainable Marine Biotechnology, Stazione Zoologica Anton Dohrn, Villa Comunale, 80112 Naples, Italy; maria.costantini@szn.it; 4 Department of Ecosustainable Marine Biotechnology, Ischia Marine Centre, Stazione Zoologica Anton Dohrn, 80077 Ischia, Italy; valerio.zupo@szn.it

**Keywords:** active biomonitoring, chemical contamination, oxidative stress biomarkers, gene expression, coastal pollution, North Ionian Sea

## Abstract

Coastal ecosystems are increasingly threatened by human activities, highlighting the need for sensitive tools to assess environmental risk. An active biomonitoring approach, using the Mediterranean mussel (*Mytilus galloprovincialis*), was employed to evaluate anthropogenic chemical contamination in the North Ionian Sea, a still poorly studied area, by comparing mussel health status before (PrePT) and after (PostPT) the peak tourist season. Bioaccumulation of metal(loid)s was quantified in whole organisms. Oxidative stress was assessed in the gills and digestive gland through catalase (CAT), superoxide dismutase (SOD), lipid peroxidation (LPO), and oxidized carbonyl proteins (OMP). Neurotoxicity was evaluated via acetylcholinesterase (AChE) activity, while gene expression of stress-related biomarkers was analysed for metallothioneins (*mt10*, *mt20*), *sod*, *cat*, Glutathione S-transferase (*gst*), and Heat Shock Protein 70 (*hsp70*). Results suggest a progressive contaminant accumulation likely associated with intensified summer anthropogenic activity. Biomarker responses revealed clear activation of oxidative stress, with tissue-specific patterns. The findings confirm the effectiveness of active biomonitoring and multibiomarker approach in assessing coastal water quality and provide valuable baseline data for the management of marine ecosystems.

## 1. Introduction

The presence of nutrients, microplastics, and organic and inorganic pollutants in aquatic environments, as a result of human activities, represents a major global urgency, with both direct and indirect impacts on overall environmental health [1,2]. Marine and coastal environments are among the most dynamic and ecologically important ecosystems, serving as vital habitats for a wide range of marine species and offering essential services to human communities [1,3,4]. Many of these compounds are persistent, leading to their accumulation in suspended matter, sediments, and marine organisms [5,6]. This is the case of heavy metal ions that, due to their toxicity, non-biodegradability, and bioaccumulative nature, pose significant risks even at trace levels [7].

Mediterranean coastal areas, including Italian shorelines [8,9], are increasingly subjected to anthropogenic pressures. This has led to increased pollutant accumulation and environmental degradation [10,11,12,13], making the Mediterranean Sea the region with the highest share of threatened habitats (32%) in the EU28 [14]. In addition, the growing attractiveness of Mediterranean coastal municipalities as summer tourist destinations [15,16] has further intensified anthropogenic pressures on these areas, leading to both direct and indirect environmental impacts. This condition is becoming particularly severe in certain regions, such as Southern Italy [9,13,17], where seasonal population surges driven by intense tourism increase environmental pressures. In these areas, pollution is exacerbated by heightened boat traffic, wastewater discharge, and littering [18]. This underscores the urgent need for targeted research programs aimed at assessing temporal and spatial variations in marine chemical contamination. In this context, the use of “sentinel organisms” offers a significant advantage over the exclusive monitoring of abiotic components, as it enables the evaluation of whether and to what extent chemical pollutants impact the physiology of marine species, with cascading effects on overall ecosystem health [11,19,20,21].

The Calabrian coast (Southern Italy), despite its considerable extension (715.7 km, about 9.7% of the Italian coastline), high biodiversity [22,23] and the presence of Sites of Community Importance (SCIs) (Natura 2000 Habitats Directive; Council Directive 92/43/EEC), has been the focus of relatively few studies investigating the impacts of anthropogenic pressures, including those associated with seasonal activities. In addition, to the best of our knowledge, and different from the higher number of research carried out on the Apulian Ionian coasts [24,25,26,27,28,29,30], only a few studies have investigated the effects of heavy metal-dependent pollution by using “sentinel organisms” along the Calabria Ionian coasts. These studies mainly focused on its central and southern areas (Roccella Ionica: [31]; Tyrrhenian and Ionian coasts of Calabria: Refs. [32,33]; Crotone: Refs. [34,35,36].

Off the northern Ionian coast of Calabria, a site of particular interest is the Amendolara Bank, designated as a Site of Community Importance SCI (SCI code IT9310053); Natura 2000 Habitats Directive; Council Directive 92/43/EEC. The coastal area (N 39°56.376′, E 16°36.887′) in front of the Amendolara Bank is characterized by dunes and low-lying zones and, in recent times, has been significantly impacted by erosion and anthropogenic factors such as urbanization and agricultural exploitation, aggravated by the lack of an adequate management policy. Moreover, the area experiences considerable pressure from mass tourism, which, as in most Calabrian coasts, is highly seasonal, with August representing the peak tourist month (Eurostat: https://ec.europa.eu/eurostat/statistics-explained/index.php?title=Tourism_statistics_-_seasonality_at_regional_level; data extracted in November 2025; accessed on 15 January 2026). This marked temporal concentration of tourism may have a severe impact on the marine environment due to the sharp increase in anthropogenic activities, including waste generation, coastal infrastructure use, and maritime traffic.

The Mediterranean mussel (*Mytilus galloprovincialis*, Lamarck, 1819) is a well-recognized bioindicator of marine contamination [11,37,38,39,40,41]. Thanks to its sessile lifestyle, filter-feeding behaviour, and ability to accumulate pollutants while triggering measurable biological responses, it is considered an important sentinel organism for environmental health assessment [11,28,42,43,44]. Moreover, its robustness and ease of transport make it ideal for active biomonitoring strategies. Within the active mussel watch framework, this involves the transplantation of organisms under controlled conditions from a reference site to selected study areas for contamination monitoring [19,45,46,47,48]. For all the above reasons, *M. galloprovincialis* represents an appropriate tool for research integrating biomarker-based assessments of molecular and cellular alterations induced by environmental pollution [38,39,49].

A primary consequence of environmental contamination by heavy metals is oxidative stress. This occurs when the production of reactive oxygen species (ROS) exceeds the neutralizing capacity of an organism’s antioxidant defence system, resulting in cellular and molecular damage. To assess oxidative stress at the cellular and tissue level, key biomarkers are used, including several enzymes such as catalase (CAT) and superoxide dismutase (SOD), which act as ROS-scavenging defence mechanisms [50,51], and Glutathione S-Transferase (GST) and acetylcholinesterase (AChE), well-recognized markers of effect and exposure, respectively [41,52]. Oxidative stress assessment also involves measuring lipid peroxidation (LPO) and protein oxidation products (OMP), indicative of molecular oxidative damage [51,53,54]. Moreover, metallothioneins (MTs), small proteins with a strong affinity for metal ions, are typically induced in response to heavy metal accumulation in biological tissues, making them reliable and selective biomarkers of metal exposure [42,55].

In the present study, we applied an active monitoring method by transplanting *M. galloprovincialis* from a farm in Crotone (Calabria, Italy) to nearshore waters off Amendolara to analyse the coastal environmental changes potentially related to anthropogenic impacts due to increased summer tourism.

The health status of mussels was assessed through a multibiomarker approach at three time points: immediately after purchase, prior to the peak tourist season, and at the end of the summer season. The study aimed to evaluate (i) bioaccumulation of contaminants (metal(loid)s in the whole organism; (ii) oxidative stress in the gills and digestive gland (DG), based on catalase (CAT) and superoxide dismutase (SOD) activities, lipid peroxidation (LPO), and protein oxidation (OMP); (iii) biomarkers of exposure (AChE activity); and (iv) expression levels of *mt10*, *mt20*, *sod*, *cat*, *gst*, and *hsp70* genes.

## 2. Materials and Methods

### 2.1. Experimental Design: Animals and Transplantation Procedure

Individuals of *Mytilus galloprovincialis* were sourced from a certified aquaculture facility in Crotone (Calabria, Italy) and transported to the study area in Amendolara (Figure S1) in cooled containers to minimise stress. Specimens within the target size range (shell length 4.5 ± 0.5 cm) were selected and cleaned to ensure the shell integrity, and only undamaged individuals were used in the experiments. Mussels were then divided into two nets, each containing 150 individuals and submerged at the exposure site in Amendolara, approximately 280 m from the shore (N 39°56.376′, E 16°36.887′) and 2 m above the seafloor. Nets were anchored to the seabed with a cotton rope and attached to a buoy to maintain neutral buoyancy. Mussel exposure began on 23 July 2023, prior to the peak of the tourist season (August), and lasted for 60 days.

Samplings were performed at three points: the first (mussels randomly sampled prior to deployment) represented the reference condition (CTRL); the second (8 days after deployment) represented the condition before the exposure to environmental conditions characterized by the peak in tourism activity (PrePT); the third (60 days after the deployment) represented the exposure to environmental conditions after the peak tourism season (PostPT). This approach follows standard active biomonitoring protocols according to [45,47].

Mussels were removed from the water, immediately placed in cool boxes and transported to the laboratory for examination. A total of 117 individuals (39 for each condition: CTRL, PrePT, and PostPT) were used. For chemical analysis, whole soft tissues were collected and stored at −20 °C. For biochemical analyses, gills and DG were dissected and stored at −80 °C.

### 2.2. Chemical Analysis

The total concentrations of twenty-eight metal(loid)s [mercury (Hg), lithium (Li), beryllium (Be), aluminium (Al), vanadium (V), chromium (Cr), manganese (Mn), iron (Fe), cobalt (Co), nickel (Ni), copper (Cu), zinc (Zn), arsenic (As), selenium (Se), strontium (Sr), silver (Ag), cadmium (Cd), antimony (Sb), lead (Pb), uranium (U), barium (Ba), bismuth (Bi), boron (B), molybdenum (Mo), rubidium (Rb), tin (Sn), thallium (Tl), and tellurium (Te)] were measured for each of the 12 samples for each condition by inductively coupled plasma mass spectrometry (ICP-MS, Aurora M90, Bruker, Billerica, MA, USA) after biomass chemical digestion. All details about chemical analyses, including sample preparation, limit of detection and limit of quantification, are reported in Supplementary Materials.

### 2.3. Acetylcholinesterase Activity

Gills and DG of 12 animals for each condition were dissected, damp-dried and homogenised in an ice-cold 100 mM phosphate buffer, pH 7.4 (1/4 *w*/*v*) with a tissue grinder. The homogenate was centrifuged at 9000× *g* at 4 °C for 30 min, and the supernatant was stored at −80 °C until biochemical measurements. The sample protein concentration was estimated according to the Bradford method by using bovine serum albumin as a standard. Aliquots of the supernatant were utilized for the spectrophotometric determination of the activity of acetylcholinesterase. Enzyme activity was measured according to Ellman et al. (1961) [56]. In a typical assay, 0.6 mg of protein was incubated at 25 °C in a final volume of 1.2 mL containing: 100 mM phosphate buffer, pH 7.4, 0.5 mM ASCh and 0.33 mM DTNB. The enzymatic reaction rate was quantified spectrophotometrically at 405 nm at both 0 and after 10 min. AChE activity expressed as nmol min^−1^ (mg of protein)^−1^. In each experiment, a blank without substrate was measured to evaluate the reaction of thiols with DTNB, and a second blank without sample was used to estimate the rate of spontaneous substrate hydrolysis.

### 2.4. Oxidative Stress Biomarkers

The evaluation of oxidative stress biomarkers (CAT, SOD, LPO, OMP) was carried out following the protocol described by Filice et al. [53]. Samples of DG and gills (*n* = 6 per condition) were homogenized in an ice-cold 100 mM Tris-HCl buffer (pH 7.2) containing a blend of protease inhibitors. A fraction of the homogenate was reserved for lipid peroxidation analysis, while the remaining fraction was centrifuged at 5000× *g* for 5 min at 4 °C. The supernatant was then used to assess protein oxidation and antioxidant enzyme activity of SOD. Protein concentration was quantified using the Bradford assay with a commercial kit (Bio-Rad Laboratories, Inc., Milan, Italy), employing bovine serum albumin (BSA) as the calibration standard.

#### 2.4.1. Antioxidant Enzyme Activity of SOD and CAT

Superoxide dismutase (SOD) activity was assessed using the pyrogallol oxidation method according to [57]. Specifically, the inhibition of pyrogallol autoxidation at pH 8.2 was measured spectrophotometrically at 420 nm and 25 °C. The reaction was conducted in a buffer containing 50 mM Tris-HCl, 1 mM EDTA, and 0.2 mM pyrogallol, with absorbance monitored every 30 s for 5 min. One unit of SOD activity was defined as the enzyme amount required to inhibit 50% of pyrogallol autoxidation. Results were expressed as U/mg of protein.

Catalase (CAT) activity was determined with a colorimetric assay kit (Thermo Fisher Scientific, Milan, Italy) following the manufacturer’s instructions, and results were expressed as U/mg of protein.

#### 2.4.2. Lipid Peroxidation

Lipid peroxidation (LPO) was evaluated by quantifying 2-thiobarbituric acid-reactive substances (TBARS), following the method of [58]. The measurement of TBARS levels was based on the detection of malondialdehyde (MDA), the primary byproduct of lipid oxidation. For this analysis, a reaction mixture was prepared by combining 0.2 mL of sample homogenate (10% *w*/*v*) in 100 mM Tris-HCl buffer (pH 7.2) with 0.2 mL of 0.8% 2-thiobarbituric acid (TBA) and 0.2 mL of 20% trichloroacetic acid (TCA). The mixture was incubated in a water bath at 100 °C for 10 min, followed by centrifugation at 7000 rpm for 10 min. The absorbance of the resulting supernatant was recorded at 540 nm to determine MDA concentration, with TBARS values expressed as MDA levels (nM) per gram of tissue, using an MDA extinction coefficient of 156,000 M^−1^ cm^−1^.

#### 2.4.3. Protein Oxidation

The levels of oxidatively modified proteins (OMP) were determined by quantifying carbonyl group content using the classic 2,4-dinitrophenylhydrazine (DNPH) assay, as described by Levine et al. (1994) [59]. Supernatant aliquots were incubated at room temperature for 1 h with 10 mM DNPH in 2 M HCl, followed by precipitation with two volumes of trichloroacetic acid (TCA). The mixture was then centrifuged at 7000 rpm for 20 min, and the resulting pellet was washed three times with a 1:1 (*v*/*v*) ethanol-ethyl acetate solution to remove excess DNPH. The pellet was subsequently dissolved in 6 M guanidine prepared in 2 N HCl. Carbonyl content was measured spectrophotometrically at 370 nm for aldehydic derivatives and 430 nm for ketonic derivatives, using an extinction coefficient of 22,000 M^−1^ cm^−1^. Results were expressed as nmol of carbonyl groups per mg of protein.

### 2.5. RNA and Protein Extraction

Following dissection, gill and DG tissues from mussels were preserved in RNAlater™ (Invitrogen, Thermo Fisher Scientific, Milan, Italy) and subsequently processed for the simultaneous extraction of RNA using the AllPrep DNA/RNA/Protein Mini Kit (Qiagen S.r.l., Milan, Italy), according to the manufacturer’s instructions. For each experimental condition, tissues collected from 9 animals were pooled into three separate pools. After the extraction procedure, RNA samples were stored under RNase-free conditions at −80 °C until further analysis. RNA concentrations were determined using a NanoDrop spectrophotometer (Thermo Fisher Scientific, Milan, Italy), and purity was assessed by measuring the λ260/λ280 absorbance ratio to evaluate potential protein contamination. The protein pellet was solubilized in 5% SDS and protein concentration was calculated by the Biuret colorimetric method (Giesse Diagnostics, Rome, Italy), according to the manufacturer’s protocol.

### 2.6. Quantitative Real-Time PCR

For each RNA extract, reverse transcription was carried out using 0.5 μg of total RNA with the iScript™ Select cDNA Synthesis Kit (Bio-Rad, Milan, Italy) and Oligo (dT) primers, following the manufacturer’s instructions. Prior to quantitative PCR (qPCR), each gene-specific primer pair was tested to verify amplification efficiency. The list of primers used is provided in Table 1. Real-time PCR reactions were performed using the SYBR™ Select Master Mix (Thermo Fisher Scientific, Milan, Italy) on a QuantStudio™ 3 Real-Time PCR System (Applied Biosystems™, Thermo Fisher, Milan, Italy), in accordance with the manufacturer’s protocol. Each reaction was carried out in duplicate in a final volume of 20 μL, containing 6 μL of DNase/RNase-free water, 1 μL of each primer (10 μM forward and reverse), and 10 μL of ready-to-use SYBR Master Mix. At the end of each amplification, melt curve analysis was performed to confirm the specificity of the PCR products (QuantStudio design & analysis software V1.6.1; Applied Biosystems™, Thermo Fisher, Milan, Italy). For normalization of gene expression data, elongation factor 1-alpha (ef1-α) and 18S rRNA were used as reference genes, as validated in previous studies (Table 1). Relative gene expression was quantified using the comparative Ct (ΔCt) method [60] (QuantStudio design & analysis software V1.6.1; Applied Biosystems™, Thermo Fisher, Milan, Italy), and results were expressed as 2^−ΔΔCt^ values, considered proportional to the abundance of the target mRNA.

### 2.7. Data Analysis

For all data, normality was assessed using the Shapiro–Wilk test, and homogeneity of variances was verified using Bartlett’s test. Since both assumptions were met (*p* > 0.05), differences between groups were analyzed using a one-way analysis of variance (ANOVA) followed by Tukey’s multiple comparisons test. Values are expressed as means ± SE (for metal(loid)s) or means ± SD (for stress biomarkers) of absolute values from individual experiments. Statistical analysis was performed by using GraphPad Prism software, version 9.00 (GraphPad Software Inc., San Diego, CA, USA).

The relationships between variables and the variation present in the dataset matrix were accounted for via biplotting both the ordination component scores and the variable loading coefficients through Principal Component Analysis (PCA) based on Pearson’s correlation matrix, in order to identify the major discriminating variables associated with a given principal component.

## 3. Results

### 3.1. Chemical Analyses

The concentrations of the analysed metal(loid)s in *M. galloprovincialis* are reported in Table 2. Overall, several elements showed detectable levels in all sampling conditions (CTRL, PrePT, and PostPT), while others (Sb, Ag, Be, Sn, Tl) were consistently below the limit of quantification (Table 2). Among the analysed elements, Zn, Fe, and Sr were found at the highest concentrations across all groups. Zinc (Zn) was the only element showing statistically significant differences among sampling times (*p* < 0.05; Table 2). In particular, Zn concentrations significantly increased in PostPT (12,929 ± 7236 μg/kg, b) compared to both CTRL (7532 ± 6886 μg/kg, a) and PrePT (8953 ± 3982 μg/kg, a), which did not differ from each other. A similar increasing trend, although not statistically significant, was observed for Fe, which rose from 2559 ± 782.7 μg/kg (CTRL) to 4109 ± 1558 μg/kg (PostPT), and for Sr, with values increasing from 1118 ± 334.6 μg/kg (CTRL) to 1541.3 ± 296.8 μg/kg (PostPT) (Table 2). Arsenic (As) was also detected at relatively high concentrations, showing a slight decrease in PrePT (931.3 ± 229.2 μg/kg) compared to CTRL (1095 ± 440.1 μg/kg), followed by an increase in PostPT (1417 ± 439.1 μg/kg). Similarly, Al concentrations increased progressively from CTRL (230 ± 61.4 μg/kg) to PostPT (718.2 ± 511.2 μg/kg), although with high variability (Table 2). Some elements, such as Cd, Co, Cr, Ni, Pb, and Cu, exhibited moderate concentrations with a general tendency to increase in PostPT compared to CTRL and PrePT. For instance, Cd increased from 52.7 ± 29.1 μg/kg (CTRL) to 84.5 ± 28.8 μg/kg (PostPT), while Ni rose from 63.3 ± 28.3 μg/kg to 98.3 ± 53.1 μg/kg. Pb and Cu also showed slight increases in PostPT (Table 2). Conversely, elements such as Mn, Mo, and Se showed variable trends without a clear pattern among the three sampling points. Mercury (Hg) concentrations remained relatively low in all conditions, although a slight increase was observed in PostPT (5.6 ± 1.8 μg/kg) compared to CTRL (3.9 ± 1.3 μg/kg). Boron (B) and lithium (Li) displayed moderate increases in PostPT compared to the other conditions, while vanadium (V) also showed a slight increase at the end of the exposure period (Table 2).

### 3.2. Biomarkers of Exposure

**Figure 1:**
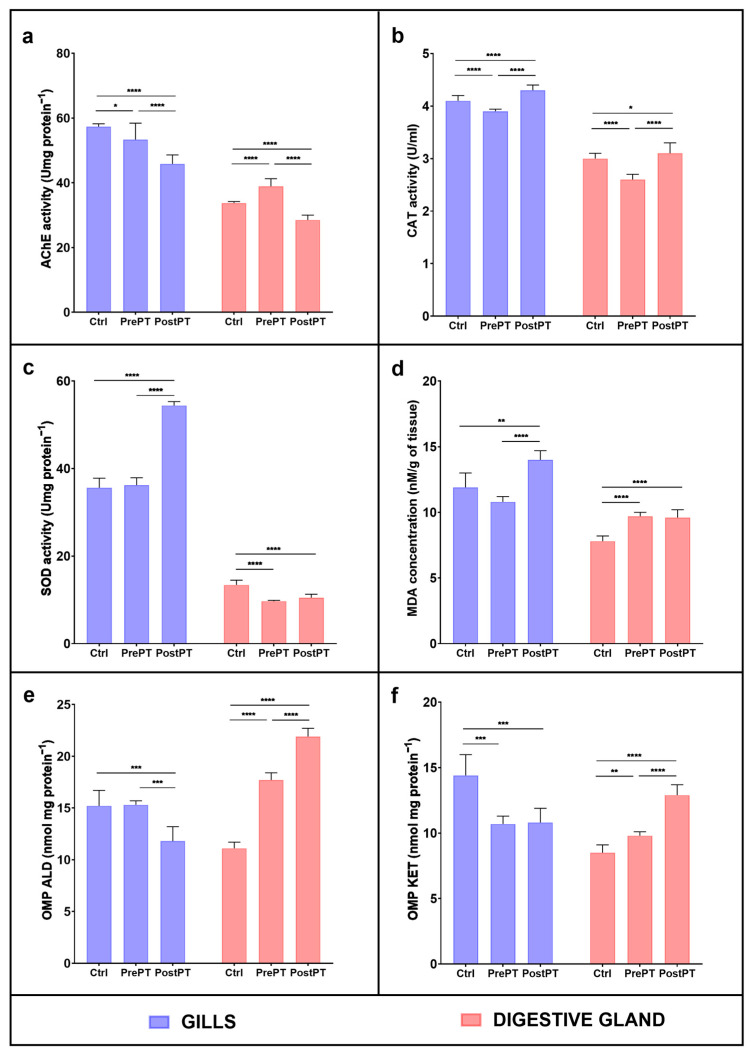
Biomarker responses in gills and digestive gland of *Mytilus galloprovincialis* under different experimental conditions (CTRL, PrePT, PostPT). The analysed biomarkers include (**a**) acetylcholinesterase (AChE), (**b**) catalase (CAT), (**c**) superoxide dismutase (SOD), (**d**) lipid peroxidation (TBARS, expressed as MDA levels), and (**e**,**f**) oxidatively modified proteins (OMP ALD and OMP CHET). Data are presented as mean ± SD (*n* = 12 for AChE and *n* = 6 for oxidative markers, for each condition). Statistical differences among groups were assessed by one-way ANOVA followed by Tukey’s post hoc test (**** *p* < 0.0001; *** *p* < 0.001; ** *p* < 0.01; * *p* < 0.05).

In the gills, a decreased AChE activity was observed at PrePT compared to CTRL, and a further decrease at PostPT compared to both CTRL and PrePT. In the DG, the trend is different, with an increase at PrePT compared to CTRL, and then an inhibition of activity at PostPT compared to both CTRL and PrePT (Figure 1a).

Activation of an oxidative response after exposure to anthropogenic impact has been evaluated by analyzing the activity of selected antioxidant enzymes (i.e., CAT and SOD), as well as by monitoring the levels of oxidation products (i.e., lipid peroxidation, LPO, and protein carbonylation) (Figure 1b–f). In particular, in both tissues, CAT activity exhibited a significant reduction at PrePT compared to CTRL, followed by a significant increase at PostPT compared to both PrePT and CTRL (Figure 1b). The activity of superoxide dismutase (SOD) in the gills exhibited higher levels in PostPT in comparison to both PrePT and CTRLs (Figure 1c). Conversely, in the digestive gland, SOD activity was significantly lower in both the PrePT and PostPT conditions compared to the CTRL group (Figure 1c).

In the gills, lipid peroxidation levels, measured as malondialdehyde (MDA) concentration, were significantly higher in PostPT than in CTRL and PrePT, while in the DG, levels were significantly higher in both PrePT and PostPT compared to CTRL (Figure 1d).

The oxidatively modified protein (OMP) levels showed a different trend in the two tissues. In the gills, aldehydic and ketonic derivatives were significantly reduced at PostPT compared to CTRL, while in the DG, both aldehydic and ketonic derivatives showed higher levels in PrePT compared to CTRL, with further increment at PostPT (Figure 1e,f).

### 3.3. Determination of Transcriptional Response by qPCR

**Figure 2:**
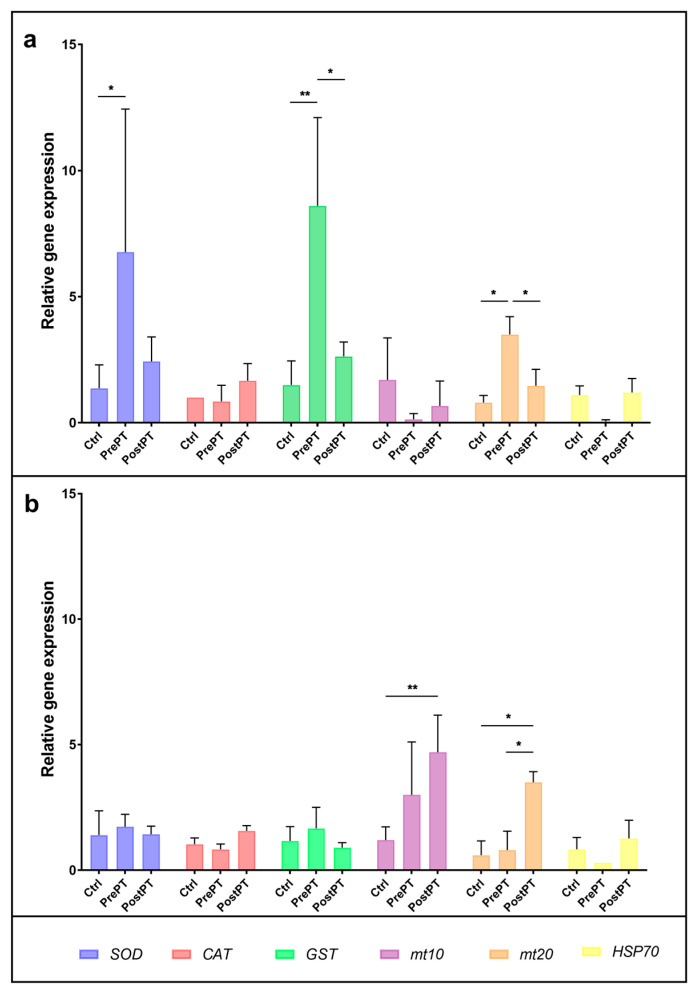
Relative gene expression (mean ± SD, *n* = 9 for each condition) of antioxidant enzyme genes (*mnsod*, *cat*, *gst*) and protein involved in the stress response (*mt10*, *mt20*, and *hsp70*) in (**a**) gills and (**b**) digestive gland from *Mytilus galloprovincialis* sampled before and after the tourist impact (PrePT and PostPT). Statistics were assessed by one-way ANOVA followed by Tukey post hoc test (** *p* < 0.01; * *p* < 0.05).

In gill tissues, a significant upregulation of *mnsod* expression was observed in the PrePT condition compared to CTRL. Similarly, *gst* expression was significantly increased in PrePT relative to CTRL. However, at PostPT, *gst* transcript levels showed a significant decrease compared to PrePT. No significant changes were observed in *cat* expression across the experimental conditions. Regarding stress-related genes in gills, *mt20* expression was significantly upregulated in PrePT compared to CTRL. This increase was followed by a significant downregulation at PostPT compared to PrePT. In contrast, no significant variations were detected for *mt10* and *hsp70* expression levels in gill tissues (Figure 2a).

In the digestive gland, gene expression patterns differed from those observed in the gills. No significant changes were detected for antioxidant-related genes (*mnsod*, *cat*, *gst*) across the experimental conditions. However, a marked transcriptional response was observed for metallothioneins. Both *mt10* and *mt20* showed a significant upregulation in PostPT compared to CTRL, with *mt20* also significantly higher than in PrePT. No significant changes in *hsp70* expression were observed (Figure 2b).

### 3.4. Multivariate Analysis (PCA)

**Figure 3:**
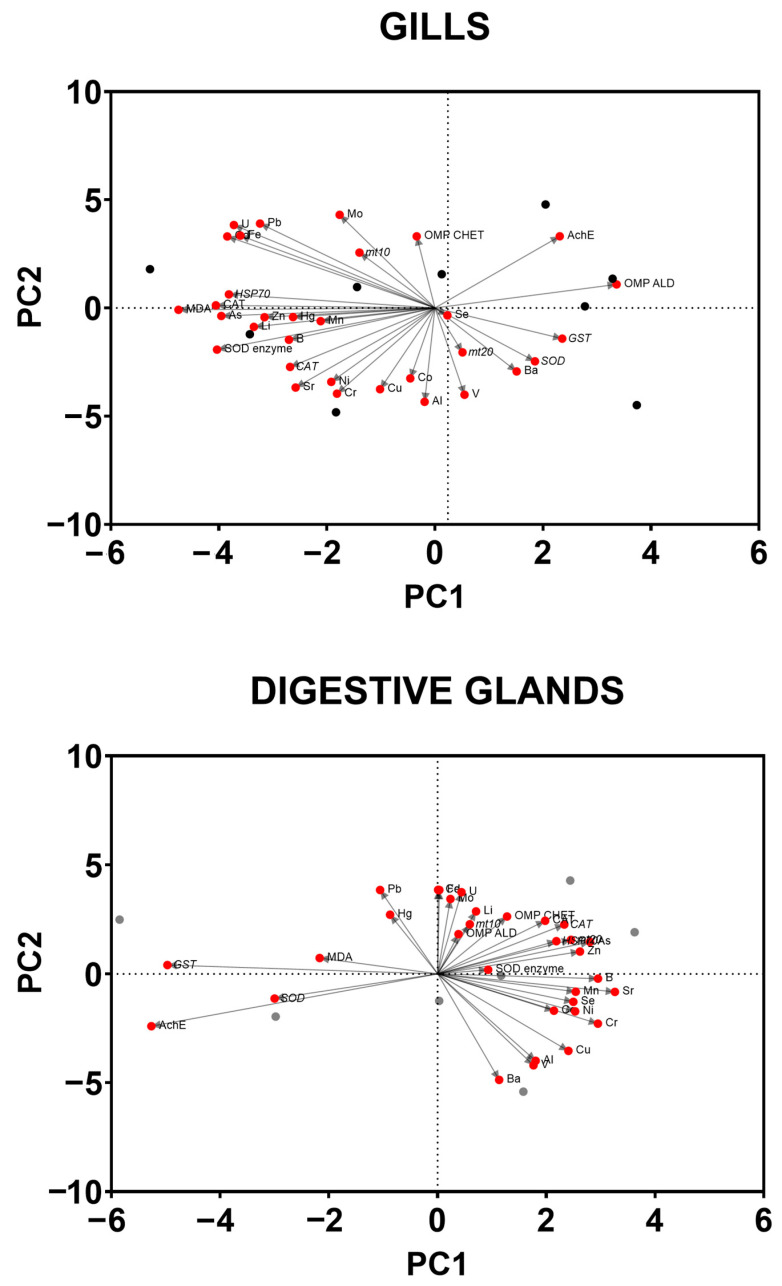
Principal Component Analysis (PCA) biplots showing the distribution of CTRL, PrePT, and PostPT samples based on metal(loid) concentrations and biomarker responses in *M. galloprovincialis*. In the gills, PC1 explained 31.91% of the total variance and the first two principal components cumulatively explained 61.68%. In the digestive gland, PC1 explained 34.39% of the variance and PC1–PC2 together explained 67.16%. Variables were standardized prior to analysis.

Principal Component Analysis (PCA) was performed to explore the overall relationships among metal(loid) concentrations and biomarker responses across sampling conditions. In gills, PC1 explained 31.91% of the total variance, while the first two principal components cumulatively explained 61.68%. In the DG, PC1 explained 34.39% of the variance and PC1–PC2 together explained 67.16%. The PCA plot showed a partial separation among CTRL, PrePT, and PostPT samples, with PostPT samples tending to cluster separately from CTRL samples along PC1 (Figure 3).

In gills, PCA showed a partial separation of PostPT samples along the component mainly associated with metal(loid)s (e.g., Zn, Fe, Pb, Ni) and oxidative stress biomarkers, particularly LPO, suggesting a relationship between contaminant accumulation and oxidative damage in this tissue. Antioxidant enzymes (CAT and SOD) were distributed along a different direction in the ordination space, consistent with their role in antioxidant defence responses rather than direct indicators of contamination. In the DG, PostPT samples clustered closer to metal(loid)s and protein oxidation biomarkers (OMP), indicating enhanced oxidative stress conditions. PrePT samples occupied an intermediate position between CTRL and PostPT groups, suggesting early physiological responses to exposure, whereas CTRL samples were associated with baseline conditions.

## 4. Discussion

Bivalve mollusks are widely used as sensitive bioindicators of marine pollution, particularly for assessing metal contamination and its biological effects through oxidative stress-related responses. Their filter-feeding activity, sedentary behavior, and capacity to bioaccumulate contaminants make them especially suitable for active biomonitoring approaches [21,52,66,67]. In this context, mussel transplantation has been extensively applied to evaluate seawater quality by monitoring seasonal variations in contaminant bioavailability and biological responses under controlled conditions [48,68]. This approach enables the standardization of biological variables such as size, age, and physiological status, thereby improving the reliability of environmental assessments.

Among the most widely used biomarkers, catalase (CAT) and superoxide dismutase (SOD) activities, lipid peroxidation (LPO, measured as MDA), and acetylcholinesterase (AChE) activity provide valuable information on oxidative stress and neurotoxicity, respectively [69,70,71]. Exposure to trace metals such as cadmium, copper, and mercury is known to induce reactive oxygen species (ROS) production, leading to lipid and protein damage in *M. galloprovincialis* [51,72]. In bivalves, the gills and DG are the primary sites of metal uptake and accumulation, respectively, reflecting different exposure pathways and physiological roles [73]. It should be noted that metal(loid) concentrations were determined in whole soft tissues, whereas biochemical biomarkers and gene expression analyses were conducted separately in the gills and DG. Therefore, tissue-specific biological responses cannot be directly associated with tissue-specific metal burdens, and the observed relationships should be interpreted cautiously.

In recent decades, coastal tourism has emerged as one of the fastest-growing economic sectors worldwide, with the Mediterranean region representing a major hotspot. This trend has raised increasing concerns regarding its environmental impact, particularly in terms of seasonal increases in pollution due to intensified maritime traffic, wastewater discharge, and coastal urbanization [15,18,74]. Within this framework, the present study provides an integrated evaluation of chemical contamination and biological responses in *M. galloprovincialis* transplanted along the northern Ionian coast of Calabria, focusing on potential environmental challenges associated with summer tourism.

The chemical analysis revealed the presence of multiple metal(loid)s in mussel tissues across all sampling periods, confirming the widespread occurrence of trace elements in the study area. Temporal fluctuations were observed for several metal(loid)s during the exposure period; however, most of these variations were not statistically significant. Zn was the only element showing a significant increase at PostPT. Therefore, the observed trends should be interpreted cautiously and may reflect the combined effects of seasonal environmental variability, exposure duration, and anthropogenic influences occurring during the summer period.

Given its extensive use in antifouling paints, urban runoff, and maritime activities, Zn is widely recognized as a tracer of anthropogenic contamination in coastal environments [75,76,77,78]. Its marked increase paves the way to the hypothesis of increased environmental pressure associated with summer tourism. The lack of statistical significance for most elements, despite observable increasing trends, may be attributed to biological variability, moderate contamination levels, and the relatively short exposure duration. Nevertheless, the overall pattern is indicative of chronic and diffuse contamination rather than acute pollution events, consistent with seasonal anthropogenic pressures typical of Mediterranean coastal systems.

The biomarker responses clearly demonstrate the activation of oxidative stress mechanisms in transplanted mussels, with distinct tissue-specific patterns. In gill tissues, a significant increase in lipid peroxidation (LPO) was observed at PostPT, indicating oxidative damage to cellular membranes. This was accompanied by an increase in antioxidant enzyme activities (CAT and SOD), suggesting an adaptive response aimed at mitigating ROS production. However, the persistence of elevated LPO levels indicates that antioxidant defenses were insufficient to fully counteract oxidative stress under prolonged exposure conditions [52,66]. In contrast, the DG exhibited a progressive increase in protein oxidation (OMP), already evident at PrePT and further enhanced at PostPT. This finding reflects the role of this tissue in detoxification and storage processes, and suggests cumulative damage associated with prolonged exposure [66,79]. The concomitant reduction in SOD activity further indicates a possible impairment of antioxidant defences under sustained stress. AChE activity showed a general inhibition at PostPT, particularly in gills, suggesting potential exposure to neurotoxic compounds. This effect may be linked not only to metals but also to complex mixtures of pollutants, including organic contaminants commonly associated with anthropogenic activities [27,80].

Gene expression analysis provided additional insights into the molecular mechanisms underlying the observed biochemical responses. In gills, the upregulation of antioxidant-related genes (*sod* and *gst*) and metallothionein (*mt20*) at PrePT indicates a rapid activation of defense mechanisms in response to initial exposure. The subsequent downregulation observed at PostPT suggests a possible exhaustion or modulation of transcriptional responses under prolonged stress conditions [39,66]. In the DG, the significant upregulation of metallothionein genes (*mt10* and *mt20*) at PostPT is consistent with the activation of metal detoxification pathways, consistent with bioaccumulation data. Metallothioneins play a key role in metal binding and sequestration, and their induction is widely recognized as a specific response to metal exposure [42,55].

Overall, these findings indicate a tissue-specific and time-dependent response, characterized by an early activation of antioxidant and detoxification pathways in the gills, followed by a later induction of metallothioneins in the digestive gland.

The multivariate PCA approach supported the presence of temporal differences among sampling conditions, although the observed separation should be interpreted cautiously due to the combined influence of seasonal variability, transplantation effects, and exposure duration (Figure 3). On the whole, PCA results suggested a tissue-specific response to environmental stress, with gills showing rapid responses to exposure and the digestive gland reflecting cumulative effects and detoxification processes. Importantly, even in the absence of widespread statistically significant differences in individual contaminants, the clear separation of PostPT samples suggests that measurable biological alterations occur in relation to seasonal environmental conditions, possibly influenced by a peak in tourism activities. As proposed by the integration of chemical and biological data, the study area resulted affected by moderate but biologically significant anthropogenic pressure, particularly during the summer tourist season. In fact, while chemical analyses alone may underestimate environmental impact, the association with biomarker responses provided sensitive early-warning signals of ecosystem stress.

### Limitations

Despite the study stressing the importance of integrating biochemical, molecular, and chemical responses in *M. galloprovincialis* under field conditions to profile environmental changes in coastal areas, a limitation remains. In fact, the lack of concurrent data, including water and sediment metal concentrations, suspended particulate matter, temperature, salinity, dissolved oxygen, and chlorophyll-a, as well as the contributions from wastewater inputs and maritime traffic, prevent from fully contextualizing the observed biological responses and from disentangling the observed effects from broader seasonal variability and other anthropogenic pressures.

## 5. Conclusions

Our findings highlight the usefulness of integrating chemical, biochemical, and molecular endpoints, supported by PCA, to explore patterns potentially associated with field exposure conditions and seasonal variability in marine environments. Results also stressed the suitability of *M. galloprovincialis* for active biomonitoring of coastal areas exposed to seasonal pressures. In the perspective of a more robust environmental analysis, and to explore the potential implications for the human population, future research should consider the advantage of correlating active biomonitoring results with abiotic evaluations, food-safety thresholds analyses, and consumer risk assessment.

## Figures and Tables

**Table 1:** Primer pair sequences, amplicon size, and GenBank accession number for qPCR analyzed genes.

Gene	RefSeq mRNA	Forward 5′–3′ Reverse 5′–3′	PCR Size	Reference
*Cat*	AY743716.2	CTCTGACCGTGGAACCCCTGA ATCACGGATGGCATAATCTGGA	193	[61]
*gst*	AF527010.1	AACTGACCACTTCAAGAATATGCC AGAAAGTCTGCCATTTACAAAGCT	127	[62]
*Mnsod*	JN863295.1	GATGCAGCAGTAGCAGTCCA GTAGGCATGCTCCCAGACAT	159	[63]
*hsp70*	AB180909.1	CGGAGGCAAGCCAAAACTAC AGCCTCGGCAGTTTCTTTCA	109	[61]
*mt10*	AY566248.1	GGGCGCCGACTGTAAATGTTC CACGTTGAAGGCCCTGTACACC	93	[64]
*mt20*	AY566247.1	TGTGAAAGTGGCTGCGGA GTACAGCCACATCCACACGC	80	[64]
*ef1-α*	AB162021.1	CCTCCCACCATCAAGACCCA GGCTGGAGCAAAGGTAACAAC	145	[65]
*18SrRNA*	JQ611492.1	GTGCTCTTGACTGAGTGTCTCG CGAGGTCCTATTCCATTATTCC	116	[65]

**Table 2:** Concentrations of metal(loid)s (μg/kg dry weight) measured in whole soft tissues of *Mytilus galloprovincialis* at the three sampling time points: control (CTRL, Day 0), before the peak of tourist impact (PrePT, Day 8), and after the peak of tourist season (PostPT, Day 60). Data are expressed as mean ± standard error (SE) *(n* = 12 for each condition). Values reported as “<” indicate concentrations below the limit of quantification (LOQ). Different letters indicate significant differences among groups according to ANOVA followed by Tukey’s post hoc test (*p* < 0.05).

	CTRL	PrePT	PostPT
Al	230 ± 61.4 ^a^	500.9 ± 550.4 ^a^	718.2 ± 511.2 ^a^
Sb	<2.0 ^a^	<2.0 ^a^	<2.0 ^a^
Ag	<2.0 ^a^	<2.0 ^a^	<2.0 ^a^
As	1095 ± 440.1 ^a^	931.3 ± 229.2 ^a^	1417 ± 439.1 ^a^
Ba	41.9 ± 15.9 ^a^	519.4 ± 1055 ^a^	42.2 ± 17 ^a^
Be	<10 ^a^	<10 ^a^	<10 ^a^
B	866 ± 291.1 ^a^	875.2 ± 365.5 ^a^	1098 ± 303.9 ^a^
Cd	52.7 ± 29.1 ^a^	49.5 ± 42.9 ^a^	84.5 ± 28.8 ^a^
Co	66.6 ± 30.5 ^a^	55.8 ± 23.2 ^a^	98.3 ± 46.3 ^a^
Cr	17.6 ± 4.3 ^a^	16 ± 9.6 ^a^	27.2 ± 10.2 ^a^
Fe	2559 ± 782 ^a^	2893 ± 1539 ^a^	4109 ± 1558 ^a^
Li	20 ± 6.3 ^a^	16.5 ± 6 ^a^	29.4 ± 7.9 ^a^
Mn	339.8 ± 159.5 ^a^	234.2 ± 124.7 ^a^	295.4 ± 98.7 ^a^
Hg	3.9 ± 1.3 ^a^	3.7 ± 1.7 ^a^	5.6 ± 1.8 ^a^
Mo	47.9 ± 17.3 ^a^	38.7 ± 17.7 ^a^	53.1 ± 34 ^a^
Ni	63.3 ± 28.3 ^a^	51.4 ± 24.1 ^a^	98.3 ± 53.1 ^a^
Pb	33.6 ± 7.5 ^a^	35.6 ± 20.3 ^a^	46.3 ± 11.4 ^a^
Cu	179.6 ± 46.8 ^a^	156.7 ± 72.3 ^a^	196.4 ± 55 ^a^
Se	195 ± 60.1 ^a^	148.1 ± 64.9 ^a^	167.6 ± 56.2 ^a^
Sr	1118 ± 334.6 ^a^	1002 ± 337.5 ^a^	1541 ± 296.8 ^a^
Sn	<20 ^a^	<20 ^a^	<20 ^a^
Tl	<2.0 ^a^	<2.0 ^a^	<2.0 ^a^
U	5.6 ± 2.1 ^a^	4.7 ± 2.8 ^a^	7.3 ± 3.6 ^a^
V	49.5 ± 19.5 ^a^	50.6 ± 37 ^a^	67 ± 37.4 ^a^
Zn	7532 ± 6886 ^a^	8953 ± 3982 ^a^	12,929 ± 7236 ^b^

## Data Availability

The original contributions presented in this study are included in the article. Further inquiries will be available on request.

## Supplementary Materials

The following supporting information can be downloaded at https://www.mdpi.com/article/10.3390/jox16030104/s1: Figure S1: The study area; Table S1: Digestion programme for total metal(loid) content determination; Table S2: Limit of Detection (LOD) and Limit of Quantification (LOQ) for the investigated elements (μg/kg).

## Abbreviations

The following abbreviations are used in this manuscript:

AChE	Acetylcholinesterase
CAT	Catalase
DG	Digestive gland
GST	Glutathione S-transferase
HSP70	Heat Shock Protein 70
LPO	Lipid peroxidation
MDA	malondialdehyde
OMP	Oxidized carbonyl proteins
PCA	Principal Component Analysis
PrePT	Pre-Peak Tourism
PostPT	Post-Peak Tourism
SOD	Superoxide Dismutase
TBARS	2-thiobarbituric acid-reactive substances

## References

[1] Saravanan P., Saravanan V., Rajeshkannan R., Arnica G., Rajasimman M., Baskar G., Pugazhendhi A. (2024). Comprehensive Review on Toxic Heavy Metals in the Aquatic System: Sources, Identification, Treatment Strategies, and Health Risk Assessment. Environ. Res..

[2] Shekhar C., Khosya R., Sharma A.K., Thakur K., Mahajan D., Kumar R., Kumar S., Sharma A.K. (2025). A Systematic Review on Health Risks of Water Pollutants: Classification, Effects and Innovative Solutions for Conservation. Toxicol. Res..

[3] El-Sharkawy M., Alotaibi M.O., Li J., Du D., Mahmoud E. (2025). Heavy Metal Pollution in Coastal Environments: Ecological Implications and Management Strategies: A Review. Sustainability.

[4] Micella I., Kroeze C., Bak M.P., Strokal M. (2024). Causes of Coastal Waters Pollution with Nutrients, Chemicals and Plastics Worldwide. Mar. Pollut. Bull..

[5] Combi T., Pintado-Herrera M.G., Lara-Martin P.A., Miserocchi S., Langone L., Guerra R. (2016). Distribution and Fate of Legacy and Emerging Contaminants along the Adriatic Sea: A Comparative Study. Environ. Pollut..

[6] Sefali S., Ruby R., Dimple D., Giri A. (2026). Toxicological Implications of Emerging Pollutants on Aquatic Organisms. Discov. Environ..

[7] Edo G.I., Samuel P.O., Oloni G.O., Ezekiel G.O., Ikpekoro V.O., Obasohan P., Ongulu J., Otunuya C.F., Opiti A.R., Ajakaye R.S. (2024). Environmental Persistence, Bioaccumulation, and Ecotoxicology of Heavy Metals. Chem. Ecol..

[8] Ausili A., Bergamin L., Romano E. (2020). Environmental Status of Italian Coastal Marine Areas Affected by Long History of Contamination. Front. Environ. Sci..

[9] Romano E., Bergamin L., Croudace I.W., Pierfranceschi G., Sesta G., Ausili A. (2021). Measuring Anthropogenic Impacts on an Industrialised Coastal Marine Area Using Chemical and Textural Signatures in Sediments: A Case Study of Augusta Harbour (Sicily, Italy). Sci. Total Environ..

[10] Brunetti L.S., Piersante C., La Russa M.F., Cellini E., Bolea E., Laborda F., Ruffolo S.A. (2025). Examining Microplastics Along the Calabrian Coastline: Analysis of Key Characteristics and Metal Contamination. Environments.

[11] Fattorini D. (2025). Bioaccumulation of Trace Elements in Mussels as Sentinels of Environmental Pollution in the Mediterranean Sea: A Review. Explor. Environ. Resour..

[12] Impellitteri F., Yunko K., Martyniuk V., Khoma V., Piccione G., Stoliar O., Faggio C. (2023). Cellular and Oxidative Stress Responses of *Mytilus galloprovincialis* to Chlorpromazine: Implications of an Antipsychotic Drug Exposure Study. Front. Physiol..

[13] Robledo Ardila P.A., Álvarez-Alonso R., Árcega-Cabrera F., Durán Valsero J.J., Morales García R., Lamas-Cosío E., Oceguera-Vargas I., DelValls A. (2024). Assessment and Review of Heavy Metals Pollution in Sediments of the Mediterranean Sea. Appl. Sci..

[14] Gubbay S., Sanders N., Haynes T., Janssen J., Rodwell J., Nieto S., García Criado M., Beal S., Borg J., Kennedy M. (2016). European Red List of Habitats. Part 1. Marine Habitats.

[15] Drius M., Bongiorni L., Depellegrin D., Menegon S., Pugnetti A., Stifter S. (2019). Tackling Challenges for Mediterranean Sustainable Coastal Tourism: An Ecosystem Service Perspective. Sci. Total Environ..

[16] Grelaud M., Ziveri P. (2020). The Generation of Marine Litter in Mediterranean Island Beaches as an Effect of Tourism and Its Mitigation. Sci. Rep..

[17] Buccolieri A., Buccolieri G., Cardellicchio N., Dell’Atti A., Di Leo A., Maci A. (2006). Heavy Metals in Marine Sediments of Taranto Gulf (Ionian Sea, Southern Italy). Mar. Chem..

[18] Mejjad N., Rossi A., Pavel A.B. (2022). The Coastal Tourism Industry in the Mediterranean: A Critical Review of the Socio-Economic and Environmental Pressures & Impacts. Tour. Manag. Perspect..

[19] Bajt O., Ramšak A., Milun V., Andral B., Romanelli G., Scarpato A., Mitrić M., Kupusović T., Kljajić Z., Angelidis M. (2019). Assessing Chemical Contamination in the Coastal Waters of the Adriatic Sea Using Active Mussel Biomonitoring with *Mytilus galloprovincialis*. Mar. Pollut. Bull..

[20] Chiesa S., Rotini A., Esposito C., Secco S., Manfra L., Trifuoggi M., Libralato G., Scalici M. (2024). Metal(Loid)s and Rare Earth Elements in *Posidonia oceanica* (L.) Delile (1813) Banquettes. Mar. Pollut. Bull..

[21] Richir J., Gobert S. (2016). Trace Elements in Marine Environments: Occurrence, Threats and Monitoring with Special Focus on the Costal Mediterranean. J. Environ. Anal. Toxicol..

[22] Marziliano P., Lombardi F., Menguzzato G., Scuderi A., Altieri V., Coletta V., Marcianò C. (2016). Biodiversity Conservation in Calabria Region (Southern Italy): Perspectives of Management in the Sites of the ‘Natura 2000’ Network. Proceedings of the International Conference on Research for Sustainable Development in Mountain Regions.

[23] Morabito A., Musarella C.M., Caruso G., Spampinato G. (2024). Biodiversity as a Tool in the Assessment of the Conservation Status of Coastal Habitats: A Case Study from Calabria (Southern Italy). Diversity.

[24] Annicchiarico C., Buonocore M., Cardellicchio N., Di Leo A., Giandomenico S., Spada L. (2011). PCBs, PAHs and Metal Contamination and Quality Index in Marine Sediments of the Taranto Gulf. Chem. Ecol..

[25] Cardellicchio N., Buccolieri A., Di Leo A., Giandomenico S., Spada L. (2008). Levels of Metals in Reared Mussels from Taranto Gulf (Ionian Sea, Southern Italy). Food Chem..

[26] Giandomenico S., Cardellicchio N., Spada L., Annicchiarico C., Di Leo A. (2016). Metals and PCB Levels in Some Edible Marine Organisms from the Ionian Sea: Dietary Intake Evaluation and Risk for Consumers. Environ. Sci. Pollut. Res..

[27] Lionetto M.G., Caricato R., Giordano M.E., Schettino T. (2004). Biomarker Application for the Study of Chemical Contamination Risk on Marine Organisms in the Taranto Marine Coastal Area. Chem. Ecol..

[28] Spada L., Annicchiarico C., Cardellicchio N., Giandomenico S., Di Leo A. (2013). Heavy Metals Monitoring in Mussels *Mytilus galloprovincialis* from the Apulian Coasts (Southern Italy). Mediterr. Mar. Sci..

[29] Storelli M.M., Storelli A., Marcotrigiano G.O. (2001). Heavy Metals in the Aquatic Environment of the Southern Adriatic Sea, Italy: Macroalgae, Sediments and Benthic Species. Environ. Int..

[30] Storelli M.M., Storelli A., Marcotrigiano G.O. (2000). Heavy Metals in Mussels (*Mytilus galloprovincialis*) from the Ionian Sea, Italy. J. Food Prot..

[31] Storelli M.M., Giacominelli-Stuffler R., Storelli A., Marcotrigiano G.O. (2005). Accumulation of Mercury, Cadmium, Lead and Arsenic in Swordfish and Bluefin Tuna from the Mediterranean Sea: A Comparative Study. Mar. Pollut. Bull..

[32] Canzanella S., Danese A., Mandato M., Lucifora G., Riverso C., Federico G., Gallo P., Esposito M. (2021). Concentrations of Trace Elements in Tissues of Loggerhead Turtles (*Caretta caretta*) from the Tyrrhenian and the Ionian Coastlines (Calabria, Italy). Environ. Sci. Pollut. Res..

[33] Esposito M., Capozzo D., Sansone D., Lucifora G., La Nucara R., Picazio G., Riverso C., Gallo P. (2020). Mercury and Cadmium in Striped Dolphins (Stenella Coeruleoalba) Stranded along the Southern Tyrrhenian and Western Ionian Coasts. Mediterr. Mar. Sci..

[34] Gallo S., Leonetti F.L., Reinero F.R., Micarelli P., Passarelli L., Giglio G., Milazzo C., Imbrogno S., Barca D., Bottaro M. (2025). Bioaccumulation Patterns in Different Tissues of Twelve Species of Elasmobranchs from the Tyrrhenian and Ionian Sea (Calabria, Southern Italy). Environments.

[35] Oliveri E., Ausili A., Barsanti M., Conte F., Delbono I., Del Core M., Giaramita L., Passaro S., Placenti F., Quinci E.M. (2022). Interferences between Natural and Anthropic Hazards in Marine-Coastal Environments: Assessing Transport from Land to the Offshore Systems in the Crotone Basin (Ionian Sea). Estuar. Coast. Shelf Sci..

[36] Amodio-Cocchieri R., Del Prete U., Arnese A., Giuliano M., Roncioni A. (1993). Heavy Metals and Polycyclic Aromatic Hydrocarbons (PAH’s) in Marine Organisms from the Ionian Sea (Italy). Bull. Environ. Contam. Toxicol..

[37] Caferro A., Iovine M.A., Impellitteri F., Faggio C., Amelio D., Gattuso A., Mileti O., Baldino N., Sperone E., Cerra M.C. (2025). Morphological and Functional Response of *Mytilus galloprovincialis* Exposed to the Enalapril Metabolite, Enalaprilat. Environ. Toxicol. Pharmacol..

[38] Chahouri A., Elazzaoui A., Lamine I., Banaoui A., Bouhaimi A., Alla A.A., Moukrim A. (2025). Bivalve Sentinels in the Central Atlantic: Unraveling Integrated Biomarker Responses in Diverse Environments. Euro-Mediterr. J. Environ. Integr..

[39] Chahouri A., Yacoubi B., Moukrim A., Banaoui A. (2023). Bivalve Molluscs as Bioindicators of Multiple Stressors in the Marine Environment: Recent Advances. Cont. Shelf Res..

[40] Multisanti C.R., Impellitteri F., Zicarelli G., Perugini M., Iovine M.A., Filice M.C., Piccione G., Imbrogno S., Faggio C. (2025). Toxicological Assessment of 2-Methylisothiazol-3(2H)-One on Physiological and Antioxidant Parameters in *Mytilus galloprovincialis*. Environ. Pollut..

[41] Perić L., Nerlović V., Žurga P., Žilić L., Ramšak A. (2017). Variations of Biomarkers Response in Mussels *Mytilus galloprovincialis* to Low, Moderate and High Concentrations of Organic Chemicals and Metals. Chemosphere.

[42] Banni M., Dondero F., Jebali J., Guerbej H., Boussetta H., Viarengo A. (2007). Assessment of Heavy Metal Contamination Using Real-Time PCR Analysis of Mussel Metallothionein Mt10 and Mt20 Expression: A Validation along the Tunisian Coast. Biomarkers.

[43] Esposito G., Mudadu A.G., Abete M.C., Pederiva S., Griglione A., Stella C., Ortu S., Bazzoni A.M., Meloni D., Squadrone S. (2021). Seasonal Accumulation of Trace Elements in Native Mediterranean Mussels (*Mytilus galloprovincialis* Lamarck, 1819) Collected in the Calich Lagoon (Sardinia, Italy). Environ. Sci. Pollut. Res..

[44] Viarengo A., Lowe D., Bolognesi C., Fabbri E., Koehler A. (2007). The Use of Biomarkers in Biomonitoring: A 2-Tier Approach Assessing the Level of Pollutant-Induced Stress Syndrome in Sentinel Organisms. Comp. Biochem. Physiol. Part C Toxicol. Pharmacol..

[45] Bocchetti R., Fattorini D., Pisanelli B., Macchia S., Oliviero L., Pilato F., Pellegrini D., Regoli F. (2008). Contaminant Accumulation and Biomarker Responses in Caged Mussels, *Mytilus galloprovincialis*, to Evaluate Bioavailability and Toxicological Effects of Remobilized Chemicals during Dredging and Disposal Operations in Harbour Areas. Aquat. Toxicol..

[46] Da Ros L., Nasci C., Marigomez I., Soto M. (2000). Biomarkers and Trace Metals in the Digestive Gland of Indigenous and Transplanted Mussels, *Mytilus galloprovincialis*, in Venice Lagoon, Italy. Mar. Environ. Res..

[47] Moschino V., Schintu M., Marrucci A., Marras B., Nesto N., Da Ros L. (2017). An Ecotoxicological Approach to Evaluate the Effects of Tourism Impacts in the Marine Protected Area of La Maddalena (Sardinia, Italy). Mar. Pollut. Bull..

[48] Roméo M., Hoarau P., Garello G., Gnassia-Barelli M., Girard J.P. (2003). Mussel Transplantation and Biomarkers as Useful Tools for Assessing Water Quality in the NW Mediterranean. Environ. Pollut..

[49] Benali I., Boutiba Z., Merabet A., Chèvre N. (2015). Integrated Use of Biomarkers and Condition Indices in Mussels (*Mytilus galloprovincialis*) for Monitoring Pollution and Development of Biomarker Index to Assess the Potential Toxic of Coastal Sites. Mar. Pollut. Bull..

[50] Lushchak V.I. (2011). Environmentally Induced Oxidative Stress in Aquatic Animals. Aquat. Toxicol..

[51] Valavanidis A., Vlahogianni T., Dassenakis M., Scoullos M. (2006). Molecular Biomarkers of Oxidative Stress in Aquatic Organisms in Relation to Toxic Environmental Pollutants. Ecotoxicol. Environ. Saf..

[52] Regoli F., Giuliani M.E. (2014). Oxidative Pathways of Chemical Toxicity and Oxidative Stress Biomarkers in Marine Organisms. Mar. Environ. Res..

[53] Filice M., Gallo S., Caferro A., Giglio G., Leonetti F.L., Milazzo C., Gattuso A., Cerra M.C., Barca D., Sperone E. (2025). Trace Element Accumulation and Oxidative Stress in Three Populations of the European Eel *Anguilla anguilla* L. from Southern Italy. Fishes.

[54] Filice M., Reinero F.R., Cerra M.C., Faggio C., Leonetti F.L., Micarelli P., Giglio G., Sperone E., Barca D., Imbrogno S. (2023). Contamination by Trace Elements and Oxidative Stress in the Skeletal Muscle of Scyliorhinus Canicula from the Central Tyrrhenian Sea. Antioxidants.

[55] Amiard J.-C., Amiard-Triquet C., Barka S., Pellerin J., Rainbow P.S. (2006). Metallothioneins in Aquatic Invertebrates: Their Role in Metal Detoxification and Their Use as Biomarkers. Aquat. Toxicol..

[56] Ellman G.L., Courtney K.D., Andres V., Featherstone R.M. (1961). A New and Rapid Colorimetric Determination of Acetylcholinesterase Activity. Biochem. Pharmacol..

[57] Tresnakova N., Famulari S., Zicarelli G., Impellitteri F., Pagano M., Presti G., Filice M., Caferro A., Gulotta E., Salvatore G. (2023). Multi-Characteristic Toxicity of Enantioselective Chiral Fungicide Tebuconazole to a Model Organism Mediterranean Mussel *Mytilus galloprovincialis* Lamarck, 1819 (Bivalve: Mytilidae). Sci. Total Environ..

[58] Tkachenko H., Grudniewska J. (2016). Evaluation of Oxidative Stress Markers in the Heart and Liver of Rainbow Trout (*Oncorhynchus mykiss* walbaum) Exposed to the Formalin. Fish Physiol. Biochem..

[59] Levine R.L., Williams J.A., Stadtman E.P., Shacter E. (1994). [37] Carbonyl Assays for Determination of Oxidatively Modified Proteins. Methods Enzymol..

[60] Schmittgen T.D., Livak K.J. (2008). Analyzing Real-Time PCR Data by the Comparative CT Method. Nat. Protoc..

[61] Giannetto A., Maisano M., Cappello T., Oliva S., Parrino V., Natalotto A., De Marco G., Fasulo S. (2017). Effects of Oxygen Availability on Oxidative Stress Biomarkers in the Mediterranean Mussel *Mytilus galloprovincialis*. Mar. Biotechnol..

[62] Boukadida K., Cachot J., Clérandeaux C., Gourves P.-Y., Banni M. (2017). Early and Efficient Induction of Antioxidant Defense System in *Mytilus galloprovincialis* Embryos Exposed to Metals and Heat Stress. Ecotoxicol. Environ. Saf..

[63] Wang Q., Yuan Z., Wu H., Liu F., Zhao J. (2013). Molecular Characterization of a Manganese Superoxide Dismutase and Copper/Zinc Superoxide Dismutase from the Mussel *Mytilus galloprovincialis*. Fish Shellfish Immunol..

[64] Dondero F., Dagnino A., Jonsson H., Caprì F., Gastaldi L., Viarengo A. (2006). Assessing the Occurrence of a Stress Syndrome in Mussels (Mytilus Edulis) Using a Combined Biomarker/Gene Expression Approach. Aquat. Toxicol..

[65] Giannetto A., Maisano M., Cappello T., Oliva S., Parrino V., Natalotto A., De Marco G., Barberi C., Romeo O., Mauceri A. (2015). Hypoxia-Inducible Factor α and Hif-Prolyl Hydroxylase Characterization and Gene Expression in Short-Time Air-Exposed *Mytilus galloprovincialis*. Mar. Biotechnol..

[66] Freitas V., Gonçalves O., Dolbeth M., Ramos S., Morais J., Ozorio R.O.d.A., Martins I., Almeida J.R. (2023). Optimization of Plastic Polymers for Shellfish Aquaculture Infrastructures: In Situ Antifouling Performance Assessment. Front. Mar. Sci..

[67] Viarengo A., Canesi L. (1991). Mussels as Biological Indicators of Pollution. Aquaculture.

[68] Capolupo M., Gunaalan K., Booth A.M., Sørensen L., Valbonesi P., Fabbri E. (2021). The Sub-Lethal Impact of Plastic and Tire Rubber Leachates on the Mediterranean Mussel *Mytilus galloprovincialis*. Environ. Pollut..

[69] Cao R., Wang D., Wei Q., Wang Q., Yang D., Liu H., Dong Z., Zhang X., Zhang Q., Zhao J. (2018). Integrative Biomarker Assessment of the Influence of Saxitoxin on Marine Bivalves: A Comparative Study of the Two Bivalve Species Oysters, Crassostrea Gigas, and Scallops, Chlamys Farreri. Front. Physiol..

[70] Klimova Y.S., Chuiko G.M., Gapeeva M.V., Pesnya D.S., Ivanova E.I. (2019). The Use of Oxidative Stress Parameters of Bivalve Mollusks Dreissena Polymorpha (Pallas, 1771) as Biomarkers for Ecotoxicological Assessment of Environment. Inland Water Biol..

[71] Perić L., Burić P. (2019). The Effect of Copper and Chlorpyrifos Co-Exposure on Biomarkers in the Marine Mussel *Mytilus galloprovincialis*. Chemosphere.

[72] Viarengo A., Canesi L., Pertica M., Poli G., Moore M.N., Orunesu M. (1990). Heavy Metal Effects on Lipid Peroxidation in the Tissues of Mytilus Gallopro Vincialis Lam. Comp. Biochem. Physiol. Part C Comp. Pharmacol..

[73] Regoli F. (1998). Trace Metals and Antioxidant Enzymes in Gills and Digestive Gland of the Mediterranean Mussel *Mytilus galloprovincialis*. Arch. Environ. Contam. Toxicol..

[74] Smiraglia D., Cavalli A., Giuliani C., Assennato F. (2023). The Increasing Coastal Urbanization in the Mediterranean Environment: The State of the Art in Italy. Land.

[75] Lenoble V., Layglon N., Pages C., D’Onofrio S., Misson B. (2026). Deciphering Copper and Zinc Leaching from Antifouling Paints with Different Operating Modes: Flux Determination and Toxicity Evidence. Mar. Pollut. Bull..

[76] Clochard M.-C., Oral O., Wade T.L., Cavani O., Castellino M., Ligiero L.M., Elan T. (2022). Zinc Detection in Oil-Polluted Marine Environment by Stripping Voltammetry with Mercury-Free Nanoporous Gold Electrode. Sci. Rep..

[77] Ramos-Filho A.M., Rodrigues P.d.A., Oliveira A.T.d., Conte-Junior C.A. (2025). A Systematic Review on Contamination of Marine Species by Chromium and Zinc: Effects on Animal Health and Risk to Consumer Health. J. Xenobiotics.

[78] Lagerström M., Lindgren J.F., Holmqvist A., Dahlström M., Ytreberg E. (2018). In Situ Release Rates of Cu and Zn from Commercial Antifouling Paints at Different Salinities. Mar. Pollut. Bull..

[79] Andrade M., Soares A.M.V.M., Solé M., Pereira E., Freitas R. (2023). Assessing the Impact of Terbium on *Mytilus galloprovincialis*: Metabolic and Oxidative Stress Responses. Chemosphere.

[80] Lionetto M., Caricato R., Calisi A., Giordano M., Schettino T. (2013). Acetylcholinesterase as a Biomarker in Environmental and Occupational Medicine: New Insights and Future Perspectives. BioMed Res. Int..

