# Supplementary material for: Tissue-Specific Biomarkers and Bioaccumulation in Mytilus galloprovincialis: Seasonal Anthropogenic Stress in the North Ionian Sea (Calabria, Italy)
Source: J Xenobiot. 2026 Jun 4;16(3):104. doi: 10.3390/jox16030104 (PMC13300931; doi:10.3390/jox16030104)
Supplement: Supplementary file 1 [file jox-16-00104-s001.zip › jox-4322621-supplementary.pdf]

Journal of
Xenobiotics

Supplementary Materials: Tissue-Specific Biomarkers and Bioaccumulation in *Mytilus galloprovincialis*: Seasonal Anthropogenic Stress in the North Ionian Sea (Calabria, Italy)

Maria Assunta Iovine, Mariacristina Filice, Luisa Albarano, Alessia Caferro, Sandra Imbrogno, Rosa Mazza, Francesca Esposito, Maria Costantini, Valerio Zupo, Alfonsina Gattuso, Giovanni Libralato and Maria Carmela Cerra

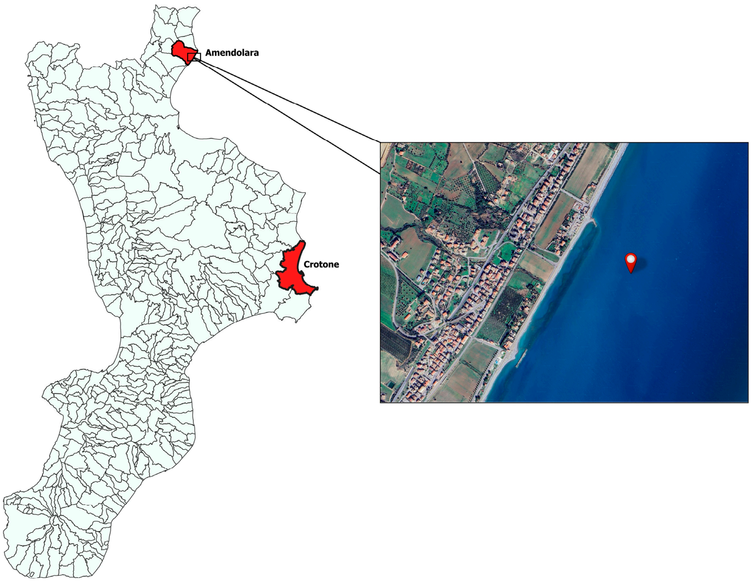

Figure S1. Study area in Calabria (Southern Italy) showing the aquaculture origin site (Crotone) and the transplantation site (Amendolara). The inset displays a satellite view of the coastal area of Amendolara, with the red marker indicating the specific offshore deployment location (located approximately 280 m from the shoreline).

Chemical analysis

Total metal(loid) content in mussel

The total concentrations of twenty-eight metal(loid)s (mercury (Hg), lithium (Li), beryllium (Be), aluminium (Al), vanadium (V), chromium (Cr), manganese (Mn), iron (Fe), cobalt (Co), nickel (Ni), copper (Cu), zinc (Zn), arsenic (As), selenium (Se), strontium (Sr), silver (Ag), cadmium (Cd), antimony (Sb), lead (Pb), uranium (U), barium (Ba), bismuth (Bi), boron (B), molybdenum (Mo), rubidium (Rb), tin (Sn), thallium (Tl), and tellurium (Te)) were measured.

Sample digestion was carried out with MARS 6 – CEM microwave digestion system. Approximately 0.250 g of tissue sample was weighed into Teflon digestion vessels, and 10 mL of ultra-pure HNO3 (67–69% v/v) together with 1 mL HF (48% v/v) were added. After a 10 min pre-reaction period at room temperature, vessels were sealed and subjected to microwave-assisted digestion using a MARS 6 – CEM system according to the program reported in Table S1. After

cooling, digested samples were filtered and quantitatively transferred into 50 mL Falcon tubes, then diluted to a final volume of 25 mL with ultrapure water (Table S1).
Sample digestion was carried out with MARS 6 – CEM microwave digestion system. Approximately 0.250 g of sample were transferred into Teflon vessels with 10 mL of ultra-pure HNO_3 (67-69% v/v) and 1 mL HF u.p. (48% v/v). After 10 minutes, the vessels were closed and transferred to the digester associated to the program shown in Table S1. After cooling, the digested samples were filtered into 50 mL falcons to a final volume of 25 mL with ultra-pure water.
All analyses were performed in triplicate to assess analytical reliability, ensuring a standard deviation (S.D.) lower than 10%. Samples were analysed by inductively coupled plasma mass spectrometry (ICP-MS, Aurora M90, Bruker, USA). Prior to the analysis, digested samples were filtered through 0.45 μm regenerated cellulose membrane filters and acidified with 3% (v/v) HNO3. Quantification was performed using external calibration curves prepared with five concentration levels for each analysed element using ICP TraceCERT® multi-element standard solutions in 5% nitric acid (Sigma-Aldrich, Milan, Italy). Ultrapure deionized water (conductivity < 0.06 μS/cm) was used for all dilutions and standard preparations. Hydrofluoric acid (HF) was included in the digestion protocol together with HNO_3 to ensure complete mineralization of residual inorganic and silicate-associated particles potentially present within whole mussel soft tissues. Since mussels were analysed without gut depuration, residual mineral particles or gut contents may have contributed to the measured concentrations of elements commonly associated with mineral phases (e.g., Al, Fe, V, Cr, and Ni).

Table S1. Digestion programme for total metal(loid) content determination.

Temp (°C)	Ramp (min)	Hold (min)	Pressure (psi)	Power (W)	Stirring
**200**	15:00	15:00	800	900-1050	Off

All plastic and glass laboratory materials used for sample preparation and chemical analyses were previously cleaned by soaking in 10% (v/v) nitric acid for at least 24 h, thoroughly rinsed with ultrapure deionized water, and dried under contamination-free conditions prior to use. Teflon digestion vessels were cleaned according to the manufacturer's recommendations between digestion cycles to minimize the risk of cross-contamination.
The Limit of Detection (LOD) and Limit of Quantification (LOQ) for each metal(loid) are reported in Table S2.

Table S2. Limit of Detection (LOD) and Limit of Quantification (LOQ) for the investigated elements (μg/kg).

Element	LOD	LOQ
Ag	0.01	0.05
Al	0.3	1
As	0.1	0.5
B	0.3	1
Ba	0.3	1
Be	0.1	0.5
Bi	0.1	0.5
Cd	0.03	0.1
Co	0.1	0.5
Cr	0.1	0.5

https://doi.org/10.3390/jox16030104

Cu	0.1	0.5
Fe	0.3	1
Hg	0.01	0.05
Li	0.1	0.5
Mn	0.1	0.5
Mo	0.1	0.5
Ni	0.1	0.5
Pb	0.1	0.5
Rb	0.1	0.5
Sb	0.1	0.5
Se	0.1	0.5
Sn	0.1	0.5
Sr	0.1	0.5
Te	0.1	0.5
Tl	0.1	0.5
U	0.1	0.5
V	0.1	0.5
Zn	0.3	1

https://doi.org/10.3390/jox16030104
